# Early and late outcomes of single versus bilateral internal thoracic artery revascularization for patients in critical condition

**DOI:** 10.1371/journal.pone.0255740

**Published:** 2021-08-05

**Authors:** Michal Fertouk, Amit Gordon, Dmitry Pevni, Tomer Ziv-Baran, Orr Sela, Rephael Mohr, Ariel Farkash, Amir Kramer, Nadav Teich, Nachum Nesher, Yanai Ben-Gal

**Affiliations:** 1 Department of Cardiothoracic Surgery, Tel-Aviv Sourasky Medical Center and Faculty of Medicine, Tel-Aviv University, Tel-Aviv, Israel; 2 Department of Epidemiology and Preventive Medicine, School of Public Health, Sackler Faculty of Medicine, Tel-Aviv University, Tel-Aviv, Israel; Ohio State University Wexner Medical Center-Department of Surgery, UNITED STATES

## Abstract

**Objective:**

The optimal surgical approach for critically ill patients with complex coronary disease remains uncertain. We compared outcomes of bilateral internal thoracic artery (BITA) versus single ITA (SITA) revascularization in critical patients.

**Methods:**

We evaluated 394 consecutive critical patients with multi-vessel disease who underwent CABG during 1996–2001. Outcomes measured were early mortality, strokes, myocardial-infarctions, sternal infections, revisions for bleeding, and late survival. The critical preoperative state was acknowledged concisely by one or more of the following: preoperative ventricular tachycardia/fibrillation, aborted sudden cardiac death, or the need for mechanical ventilation or for preoperative insertion of intra-aortic-balloon counter-pulsation.

**Results:**

During the study period, 193 of our patients who underwent SITA and 201 who underwent BITA were in critical condition. The SITA group was older (mean 68.0 vs. 63.3 years, p = 0.001) and higher proportions were females (28.5% vs. 18.9% p = 0.025), after recent-MI (69.9% vs. 57.2% p = 0.009) and with left-main disease (38.3% vs. 49.3% p = .029); the median logistic EuroSCORE was higher (0.2898 vs. 0.1597, p<0.001). No statistically significant differences were observed between the SITA and BITA groups in 30-day mortality; and in rates of early CVA, MI and sternal infections (13.0% vs. 8.5%, p = 0.148; 4.1% vs. 6.0%, p = 0.49; 6.7% vs. 4.5%, p = 0.32 and 2.1% vs. 2.5%, p>0.99, respectively). Long-term survival (median follow-up of 15 years, interquartile-range: 13.57–15) was better in the BITA group (median 14.39 vs. 9.31± 0.9 years, p = 0.001). Propensity-score matching (132 matched pairs) also yielded similar early outcomes and improved long-term survival (median follow-up of 15 years, interquartile-range: 13.56–15) for the BITA group (median 12.49±1.71 vs. 7.63±0.99 years, p = 0.002). In multivariable analysis, BITA revascularization was found to be a predictor for improved survival (hazard-ratio of 0.419, 95%CI 0.23–0.76, p = 0.004).

**Conclusions:**

This study demonstrated long-term survival benefit for BITA revascularization in patients in a critical pre-operative state who presented for surgical revascularization.

## Introduction

Surgical revascularization with the left internal thoracic artery (ITA) to the left anterior descending artery is an established therapeutic strategy to prolong life in patients with severe coronary artery disease [1]. Over the years, a few retrospective studies have demonstrated improved outcomes with multiple arterial grafts and most definitely with bilateral ITA (BITA) revascularization [2–6]. Moreover, two meta-analyses demonstrated a distinctive survival benefit and less reinterventions for BITA vs single ITA (SITA) revascularization strategy [7, 8]. The ART trial validated a clinical benefit for multi-arterial versus SITA revascularization [9], and a designated randomized trial assessing the true prognostic consequence of BITA vs SITA is currently ongoing [10]. All these reports focused mostly on preselected healthy and comparatively young and stable individuals with relatively protracted life expectancy. However, the data are very limited regarding the true implication of BITA revascularization in persons with multi-vessel disease who present for surgical revascularization while in critical condition.

In our center, skeletonized BITA grafting is the routine approach for most patients in need of primary coronary artery bypass grafting (CABG), even in the presence of multiple risk factors, older age and comorbidities. Patients with presumed lower life expectancy and pronounced comorbidities are not ruled out from receiving BITA revascularization procedures [11]. This is despite the predicament regarding accompanying risk factors for deep sternal wound infections (DSWI), and most particularly in regard to patients with chronic obstructive pulmonary disease (COPD) and women with obesity and diabetes mellitus [12, 13]. In this report, we investigated early and late outcomes of SITA versus BITA revascularization procedures performed in critical patients.

## Materials and methods

Because the research was a retrospective data research that was analyzed anonymously, and with no patients involvement, the Tel Aviv Sourasky Medical Center Institutional Review Board (Helsinki Committee) granted permission to not ask for consent. This study encompassed all the critical patients with multi-vessel coronary artery disease who underwent isolated CABG in Tel-Aviv Sourasky Medical Center during 1996–2011, deploying either a single skeletonized ITA to the left anterior descending artery along with other conduits or a bilateral skeletonized ITA to the anterior and lateral walls. BITA revascularization was performed with the "composite" configuration, in which a free right ITA is attached proximally as a composite Y graft to the left ITA, or by the "in situ" configuration with an in situ right ITA supporting the LAD and an in situ left ITA to the lateral wall [11]. Importantly, for the purpose of this study, the critical preoperative state was acknowledged in any one or more of the following: preoperative ventricular tachycardia or fibrillation, aborted sudden death, preoperative ventilation before arrival in the anesthetic room and pre-operative insertion of an intra-aortic balloon counter-pulsation (IABP). Of note, evolving myocardial infarction (MI), the need for inotropic or vasopressor therapy, and preoperative acute renal failure (anuria or oliguria < 10 ml/hr.) were not part of the study’s inclusion criteria due to unavailable, unsubstantiated or insufficient data. The study was approved by the Tel Aviv Sourasky Medical Center Institutional Review Board (Helsinki Committee), and a waiver for informed consent was granted due to the retrospective study design.

The decision to perform either BITA or SITA grafting was essentially made according to each surgeon’s discretion. BITA revascularization is the preferred revascularization strategy in our center also in complex and older patients [14]. All ITAs were harvested as skeletonized vessels [13]. Further technical aspects of the BITA procedures are detailed in previous publications of our group [15].

EuroSCORE clinical data standards were used to analyze patient data [16]. The definition of early mortality was mortality within the index revascularization or the first month from surgery. Cerebrovascular accident was considered as a new long-lasting neurological impairment with established evidence by computed tomography. The definition of DSWI necessitated an evident sternal or peri-sternal infection (with clinical and laboratory evidence) that required an open surgical intervention. An emergent operation was defined as an operation performed within 24h of catheterization or in patients with evident pre-operative acute or evolving MI, pulmonary edema or cardiogenic shock.

Early outcomes from the index hospitalizations were extracted from patients’ medical records, discharge letters and department databases. We obtained information regarding late mortality of our patients from the Israeli National Registry database.

### Statistical analysis

Categorical variables were described as frequencies and percentages. Continuous variables were evaluated for normal distribution using histograms, and reported as means and standard deviations, or medians and interquartile ranges. The Chi-square test was used to compare categorical variables between the two configurations, and the independent samples T-test and Mann-Whitney tests were applied to compare continuous variables. The length of follow-up was observed using the reverse censoring method. Kaplan Meier curve analysis was used to describe survival during the follow-up period and to report median survival time. The log-rank test was used to compare survival between the two techniques. Multivariate Cox regression was applied to evaluate the association between 15-year mortality and configuration technique, while controlling for possible confounders. Each regression contained four blocks. In the first block, configuration technique, age and gender were forced into the regression. In the second and third blocks, pre-operative and operative parameters were included as potential variables for inclusion in the model using the backward method (the Wald test was used and p>0.1 was the criteria for removal). Finally, surgical era was forced into the regression in the fourth block. The two groups were matched according to the probability of a patient undergoing CABG using BITA. The probability (propensity score) was calculated using a logistic regression model. The following parameters were used to calculate the propensity score: sex, age, non-insulin dependent diabetes mellitus, insulin-dependent diabetes mellitus, diabetes mellitus end-organ damage, chronic obstructive pulmonary disease, chronic heart failure, chronic renal failure, recent MI (less than 3 weeks), old MI, unstable angina pectoris, ejection fraction <30%, the use of an IABP, emergency redo procedures, peripheral vascular disease, number of vessel disease, left main disease and percutaneous transluminal coronary angioplasty. An absolute difference in the propensity score of up to 5% was considered acceptable for matching. Standardized differences were calculated to compare the two groups before and after matching. The matched cohorts were compared using the McNamar test for the categorical variables, and the paired T-test and Wilcoxon test for the continuous variables. Stratified Cox regression was used to compare survival between the two matched groups. All statistical tests were two sided and p<0.05 was considered statistically significant. Statistical analysis was performed with SPSS statistical software (IBM SPSS Statistics for Windows, version 27, IBM Corp., Armonk, NY, USA, 2020).

## Results

### Unmatched cohort

This study included 394 critically defined patients, 201 operated with BITA to the left side and 193 who were operated by SITA and additional grafts (mostly saphenous vein grafts). The pre-procedural and intra-intraoperative characteristics are summarized in Table 1. Compared to the BITA group, the mean age of the SITA group was older, and the proportions were greater of women, and of patients after recent MIs (less than 3 weeks), acute MIs, redo procedures and left main disease. Logistic EuroSCORE levels were higher in the SITA than the BITA group.

**Table 1:** Preoperative and intraoperative characteristics of critical patients who underwent SITA and BITA grafting revascularizations—Unmatched and matched cohorts.

	All	Unmatched cohort n(%)				Matched cohort n(%)			
		SITA	BITA	P value	Standardized Difference	SITA	BITA	P value	Standardized Difference
	n = 394	n = 193	n = 201			n = 132	n = 132		
**Male**	301(76.4%)	138(71.5%)	163(81.1%)	0.025^Ch^	-0.227	95(72%)	102(77.3%)	0.41^MN^	-0.122
**Age(years), mean(SD)**	65.65(11.53)	68.05(10.64)	63.33(11.89)	<0.001^IT^	0.418	67.64(10.72)	66.24(10.88)	0.254^PT^	0.13
**NIDDM**	128(32.5%)	70(36.3%)	58(28.9%)	0.116^Ch^	0.159	39(29.5%)	46(34.8%)	0.419^MN^	-0.114
**IDDM**	16(4.1%)	11(5.7%)	5(2.5%)	0.106^Ch^	0.163	6(4.5.0%)	3(2.3%)	0.508^MN^	0.125
**COPD**	37(9.4%)	21(10.9%)	16(8.0%)	0.32^Ch^	0.1	15(11.4%)	12(9.1%)	0.648^MN^	0.075
**CHF**	141(35.8%)	78(40.4%)	63(31.3%)	0.06^Ch^	0.19	45(34.1%)	45(34.1%)	>0.999^MN^	0
**CRF**	40(10.2%)	24(12.4%)	16(8.0%)	0.141^Ch^	0.148	17(12.9%)	12(9.1%)	0.424^MN^	0.121
**Recent MI**	250(63.5%	135(69.9%)	115(57.2%)	0.009^Ch^	0.267	84(63.6%)	83(62.9%)	>0.999^MN^	0.016
**Age>70 years**	154(39.1%)	83(43%)	71(35.3%)	0.118^Ch^	0.158	57(43.2%)	58(43.9%)	>0.999^MN^	-0.015
**Old MI**	133(33.8%)	71(36.8%)	62(30.8%)	0.212^Ch^	0.126	47(35.6%)	44(33.3%)	0.791^MN^	0.048
**Acute MI**	214(54.3%)	122(63.2%)	92(45.8%)	0.001^Ch^	0.356	75(56.8%)	68(51.5%)	0.45^MN^	0.107
**Unstable Angina**	236(59.9%)	116(60.1%)	120(59.7%)	0.935^Ch^	0.008	77(58.3%)	79(59.8%)	0.89^MN^	-0.031
**EF<30%**	94(23.9%)	63(32.6%)	31(15.4%)	<0.001^Ch^	0.411	27(20.5%)	27(20.5%)	>0.999^MN^	0
**IABP**	326(82.7%)	154(79.8%)	172(85.6%)	0.129^Ch^	-0.153	110(83.3%)	109(82.6%)	>0.999^MN^	0.02
**ReDo**	24(6.1%)	18(9.3%)	6(3.0%)	0.009^Ch^	0.266	5(3.8%)	6(4.5%)	>0.999^MN^	-0.038
**PVD**	82(20.8%)	40(20.7%)	42(20.9%)	0.967^Ch^	-0.004	32(24.2%)	29(22%)	0.766^MN^	0.054
**NOVS**	291(73.9%)	139(72%)	152(75.6%)	0.416^Ch^	-0.082	99(75.0%)	96(72.7%)	0.78^MN^	0.052
**Left Main disease**	173(43.9%)	74(38.3%)	99(49.3%)	0.029^Ch^	-0.221	59(44.7%)	55(41.7%)	0.699^MN^	0.061
**Prior PCI**	80(20.3%)	47(24.4%)	33(16.4%)	0.05^Ch^	0.198	28(21.2%)	27(20.5%)	>0.999^MN^	0.019
**EuroSCORE, median(IQR)**	11(9–14)	12(9.5–15)	10(8–12)	<0.001^MW^	0.592	12(9–14)	10(9–13)	0.052^WL^	0.233
**Logistic, median(IQR)**	0.2023(0.1086–0.4082)	0.2898(0.1454–0.5013)	0.1597(0.0955–0.2885)	<0.001^MW^	0.391	0.2578(0.1218–0.4646)	0.181(0.1094–0.3602)	0.036^WL^	0.275
**Bypass No.≥3**	254(64.5%)	116(60.1%)	138(68.7%)	0.076^Ch^	-0.179	80(60.6%)	87(65.9%)	0.427^MN^	-0.11
**OPCAB**	85(21.6)	41(21.2%)	44(21.9%)	0.876^Ch^	-0.016	25(18.9%)	33(25%)	0.28^MN^	-0.147
**Patients operated after the year 2000**	241(61.2%)	148(76.7%)	93(46.3%)	<0.001^Ch^	0.658	103(78%)	62(47%)	<0.001^MN^	2.242

SITA: single internal thoracic artery revascularization, BITA: bilateral internal thoracic artery revascularization, SD: standard deviation, IQR: interquartile ratio, NIDDM: non-insulin-dependent diabetes mellitus, IDDM, insulin-dependent diabetes mellitus, COPD: chronic obstructive pulmonary disease, CHF: congestive heart failure, CRF: chronic renal failure, MI: myocardial infarction, EF: ejection fraction, IABP: intra-aortic balloon counter-pulsation, ReDo: redo operation, PVD: peripheral vascular disease NOVS: number of vessel disease, PCI: percutaneous coronary intervention, OPCAB: off-pump coronary artery bypass; IT-Independent samples t-test; PT-Paired t-test; Ch-Chi square test; MN-McNemar’s test; MW-Mann-Whitney test; WL-Wilcoxon test

Mortality did not differ significantly (13% for the SITA vs. 8.5% for the BITA group, p = 0.148), nor did other early measurable outcomes (Table 2).

**Table 2:** Early outcomes of SITA and BITA grafting revascularizations in critical patients—Unmatched and matched cohorts.

	All	Unmatched cohort n (%)			Matched cohort n (%)		
		SITA	BITA	P value	SITA	BITA	P value
	n = 394	n = 193	n = 201		n = 132	n = 132	
**Early mortality**	42 (10.7%)	25 (13%)	17 (8.5%)	0.148^Ch^	23 (17.4%)	15 (11.4%)	0.215^MN^
**Deep infection**	9 (2.3%)	4 (2.1%)	5 (2.5%)	>0.999^F^	3 (2.3%)	4 (3.0%)	>0.999^MN^
**Perioperative CVA**	20 (5.1%)	8 (4.1%)	12 (6.0%)	0.409^Ch^	6 (4.5%)	7 (5.3%)	>0.999^MN^
**Perioperative MI**	22 (5.6%)	13 (6.7%)	9 (4.5%)	0.329^Ch^	8 (6.1%)	8 (6.1%)	>0.999^MN^
**Revision for bleeding**	14 (3.6%)	7 (3.6%)	7 (3.5%)	0.938^Ch^	5 (3.8%)	5 (3.8%)	>0.999^MN^

SITA: single internal thoracic artery revascularization, BITA: bilateral internal thoracic artery revascularization CVA: cerebrovascular accident, MI: myocardial infarction; Ch-Chi square test; MN-McNemar’s test; F-Fisher’s exact test.

**Fig 1:**
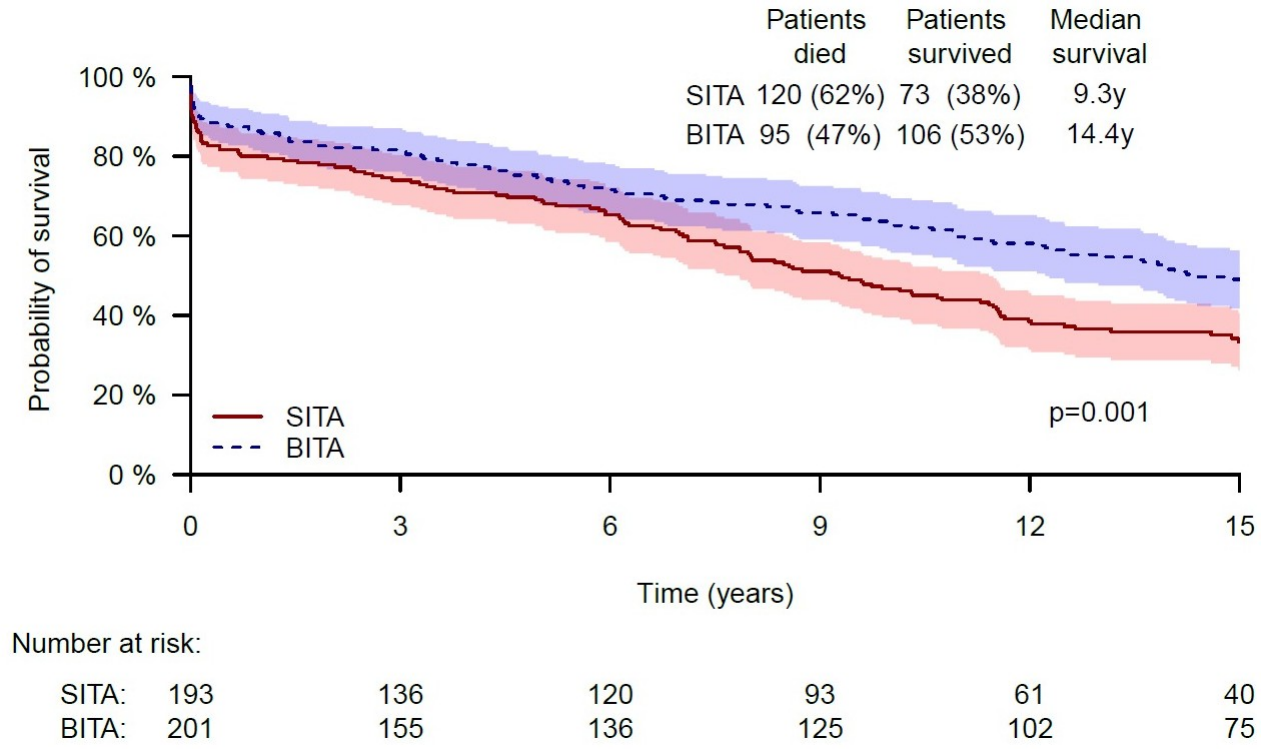
Kaplan-Meier curves of SITA and BITA grafting revascularizations in critical patients—Unmatched cohort.

After a follow up of up to 15 years (median 15, interquartile range: 13.57–15), a significant survival advantage was observed in the BITA versus the SITA group, as depicted by the Kaplan–Meier survival curves (Fig 1). The 5, 10 and 15-year survival rates were 74.2% vs. 69.1%, 63.1% vs. 46.7% and 49.0% vs. 33.4%, respectively, p = 0.001. However, in multivariable analysis, the revascularization modality (SITA vs. BITA) was not found to affect survival in the unmatched cohort (hazard ratio [HR] = 0.87 (95%CI 0.629–1.203, p = 0.399). The following variables were significant for increased mortality: older age, non-insulin-dependent diabetes mellitus, end-organ damage following diabetes mellitus, COPD, recent MI, redo operation and the number of vessels that were revascularized. The respective HRs were 1.056 (95%CI 1.041–1.072, p<0.001), 1.375 (95%CI 1.02–1.852, p = 0.037), 1.892 (95%CI 1.238–2.891, p = 0.003), 1.984 (95%CI 1.315–2.996, p = 0.001), 1.518 (95%CI 1.118–2.06, p = 0.007), 1.787 (95%CI 1.012–3.156, p = 0.045) and 1.775 (95%CI 1.219–2.584, p = 0.003).

### Matched cohort

After propensity matching, we isolated 132 pairs of comparable patients with a median follow-up of 15 years (interquartile range: 13.56–15). Significant differences were not found between the matched groups in any of the examined characteristics, except for a small but significantly higher proportion of patients in the SITA group with a high logistic EuroSCORE, and a higher proportion operated after the year 2000 (Table 1). As with the unmatched cohort, postoperative differences were not found between the matched BITA and SITA groups in early mortality (11.4% and 17.4%, respectively, p = 0.215) nor in other measurable outcomes such as perioperative strokes, MIs, revisions for bleeding and DSWI (Table 2).

**Fig 2:**
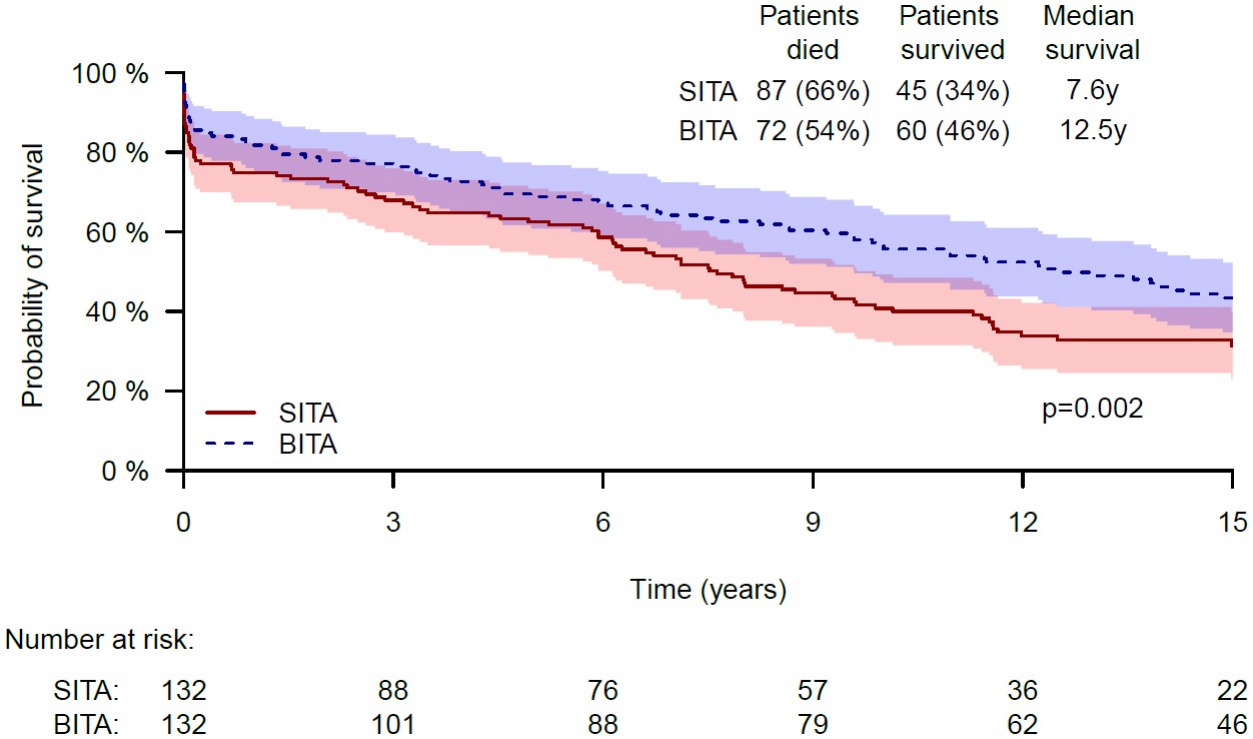
Kaplan-Meier curves of SITA and BITA grafting revascularizations in critical patients—Unmatched cohort.

Despite the above, a significant difference was found in long-term survival, in favor of the BITA vs. SITA group, as depicted in the Kaplan–Meier survival curves (Fig 2). The respective 5, 10 and 15-year survival rates were 68.8% vs. 62.5%, 56.5% vs. 40.8% and 43.5% vs. 31.4%, p = 0.002. In multivariable analysis of the matched groups, BITA revascularization was found to be associated with better survival, with a hazard ratio of 0.419 (95%CI 0.232–0.757, p = 0.004). As in the unmatched cohort, in the matched cohort additional variables were found significant for increased mortality: end-organ damage following diabetes mellitus, old MI and the number of vessels that were revascularized. The respective HRs were 3.995 (95%CI 1.221–13.074, p = 0.022), 0.313 (95%CI 0.135–0.727, p = 0.007) and 5.95 (95%CI 2.455–14.425, p<0.001).

## Comment

A few studies [2–6, 17–19], together with two historic meta-analyses [7, 8], exhibited better late outcome among patients with multi-vessel coronary artery disease who underwent surgical myocardial revascularization that deployed BITA rather than SITA strategy. While the recent ART trial demonstrated superiority of multiple arterial grafts over SITA revascularization [9], a randomized trial is currently evaluating the benefit of BITA revascularization [10]. However, most of these studies examined patients with anticipated prolonged life expectancy. These included most frequently young males, in a relatively healthy condition without severe comorbidities such as non-insulin-dependent diabetes mellitus, low left ventricular ejection fraction, recent MI, COPD, peripheral vascular disease and chronic renal failure. Moreover, BITA revascularization was rarely deployed in patients in need of emergent surgical intervention [2, 7].

A study by Schumer et al. [20] evaluated 212 patients who underwent emergent surgical revascularization among a total of 5,940 CABG procedures. Emergent patients were considered as those operated during an "evolving MI, shock (including the need for IABP insertion prior to surgery), ongoing ischemia, angiographic accident and other". In that study, patients classified as emergent versus non-emergent had significantly higher rates of early mortality (6.6% vs. 1.3%), postoperative need for IABP, bleeding, dialysis and prolonged length of stay. Notably, BITA revascularization was not part of the analysis, and the inclusion criteria for the emergent group were quite different from those of the current study.

A previous study from this group [21] evaluated the outcome of 215 patients, with the single inclusion criteria of preoperative insertion of an IABP between 1996 and 2001. The analysis revealed associations with reduced midterm (8 years) survival of the following: peripheral vascular disease, off-pump coronary artery bypass surgery, advanced age, prolonged cardiopulmonary bypass time, female gender and a decreased number of grafts. Although BITA revascularization was not scrutinized as part of the Cox analysis in that study, BITA demonstrated better midterm survival than SITA: 62.6% vs. 39.5%, p<0.001.

A retrospective analysis [22] of emergent or salvage coronary revascularization procedures at four northern European university hospitals included 614 patients with a preoperative state of acute coronary syndrome with ST elevation MI (38%), non-ST elevation MI (47%), and in need of inotropic drugs (14%) or a preoperative IABP insertion (13%). Eight percent of the patients endured cardiopulmonary resuscitation and 1% needed a cardiac massage during sternotomy. Hospital mortality was 13% and 41%, and five-year survival 79% and 15%, for the emergent and the salvage patients, respectively. While that study indicates the observable surgical challenge in critical patients, deducing a definite conclusion on the current analysis and revascularization strategies is difficult, for a number of reasons. For one, the heterogeneous features of that cohort were remarkable, both inherently and also compared to the current analysis. Moreover, BITA revascularization procedures were not performed in that study; 20% of the patients were operated exclusively with saphenous vein grafts and the salvage group included 53% non-ITA CABG procedures.

A few reports, including from our group, have focused on patients with acute or recent MIs [23–27] while assessing the outcomes and feasibility of arterial and BITA revascularization procedures or various surgical strategies such as off-pump or beating-heart on-pump procedures. Again, all the studies that focused on patient populations during or shortly after an ischemic myocardial insult differed from the current study in inclusion criteria.

In this study we evaluated long term outcome (up to 15 years) of 394 patients who underwent isolated surgical revascularization procedures deploying either single or bilateral ITAs in the setting of a critical state. The latter was determined by preoperative ventricular dysrhythmia, aborted sudden cardiac death, resuscitation, ventilation or the need for IABP insertion. We further examined these two revascularization strategies on two matched groups of 132 patients each. The high early mortality rate presented in this study (10.7% for the whole cohort) substantiates the authenticity of these patients being critically ill and in severe condition. Nonetheless, the criteria selected for this study differ from those of previous studies that evaluated high-risk patients, and that were based mostly on myocardial ischemia or infarction [23–30]. The two most important findings of the current study are the short-term safety (equal early mortality and morbidity for the two revascularization strategies), together with the long-term survival benefit gained by deploying the BITA revascularization strategy in high-risk patients.

In general, high-risk patients are not indiscriminately designated for SITA revascularization in our center, even in the face of serious comorbidities. On the contrary, our group’s predisposition has been to routinely perform left-sided BITA revascularization for most patients. Consequently, surgeons who contributed to the current study were at large highly skilled in skeletonized ITA harvesting (for extra graft length) and in all technical aspects, strategies and configurations of BITA grafting that were previously described [11–13, 15, 31]. This familiarity with BITA skeletonization and configuration techniques should be noted as a clear prerequisite for the appropriateness and safety of BITA grafting in these high-risk patients.

The single-center retrospective observational design of this study bears some characteristic limitations. First and foremost, the definition used to classify a patient as critical may differ from that of other studies. Notably, in this study the definition did not include a few key elements such as clear ischemic insult, the need for inotropic or vasopressor support, and an acute preoperative oliguric state. As such, all assumptions and conclusions might be compromised if not considered in the exact context of our definition of a critical state. Moreover, the inclusion criteria selected may have caused an inevitable disparity between the patients included in the study, as the operative risk for a post resuscitated patient can differ substantially from that of a patient in whom an IABP was inserted as a preventive measure due to a highly disturbing coronary anatomy. In addition, the late outcome analysis focused merely on survival, while important information regarding late major adverse cardiac and cerebrovascular events was not available for all patients. Another limitation results from the prolonged time span, notably, a possible calendar bias. This was addressed by the inclusion of the surgical era (operations before or after the year 2000) within the multivariable analysis, as a possible confounder. Additionally, the surgical strategy executed i.e., the use of either SITA or BITA revascularization was determined by each surgeon, subjected to personal clinical judgement, and sometimes taken with unspecified or ambiguous reasons, without strict guidelines that dictate the predilection of either strategy. This, together with the general tendency to avoid BITA revascularization in patients with increased risk for DSWI, may have led to a treatment allocation bias.

In conclusion, this study showed comparable early outcomes, and improved long-term survival, following isolated CABG with BITA compared to SITA revascularization in patients with preoperative ventricular dysrhythmias, resuscitation, in need for preoperative mechanical ventilation or preoperative insertion of an IABP. In multivariate analysis, BITA strategy was found to be an independent predictor for long-term survival. Further studies are needed to determine the exact role of BITA revascularization in these high-risk patients.
